# Kv7 Channels in Lung Diseases

**DOI:** 10.3389/fphys.2020.00634

**Published:** 2020-06-26

**Authors:** Gema Mondejar-Parreño, Francisco Perez-Vizcaino, Angel Cogolludo

**Affiliations:** ^1^Departamento de Farmacología y Toxicología, Facultad de Medicina, Universidad Complutense de Madrid, Madrid, Spain; ^2^Ciber Enfermedades Respiratorias (Ciberes), Madrid, Spain; ^3^Instituto de Investigación Sanitaria Gregorio Marañón (IISGM), Madrid, Spain

**Keywords:** Kv7 channels, KCNE, respiratory diseases, asthma, chronic obstructive pulmonary disease, pulmonary hypertension

## Abstract

Lung diseases constitute a global health concern causing disability. According to WHO in 2016, respiratory diseases accounted for 24% of world population mortality, the second cause of death after cardiovascular diseases. The Kv7 channels family is a group of voltage-dependent K^+^ channels (Kv) encoded by *KCNQ* genes that are involved in various physiological functions in numerous cell types, especially, cardiac myocytes, smooth muscle cells, neurons, and epithelial cells. Kv7 channel α-subunits are regulated by KCNE1–5 ancillary β-subunits, which modulate several characteristics of Kv7 channels such as biophysical properties, cell-location, channel trafficking, and pharmacological sensitivity. Kv7 channels are mainly expressed in two large groups of lung tissues: pulmonary arteries (PAs) and bronchial tubes. In PA, Kv7 channels are expressed in pulmonary artery smooth muscle cells (PASMCs); while in the airway (trachea, bronchus, and bronchioles), Kv7 channels are expressed in airway smooth muscle cells (ASMCs), airway epithelial cells (AEPs), and vagal airway C-fibers (VACFs). The functional role of Kv7 channels may vary depending on the cell type. Several studies have demonstrated that the impairment of Kv7 channel has a strong impact on pulmonary physiology contributing to the pathophysiology of different respiratory diseases such as cystic fibrosis, asthma, chronic obstructive pulmonary disease, chronic coughing, lung cancer, and pulmonary hypertension. Kv7 channels are now recognized as playing relevant physiological roles in many tissues, which have encouraged the search for Kv7 channel modulators with potential therapeutic use in many diseases including those affecting the lung. Modulation of Kv7 channels has been proposed to provide beneficial effects in a number of lung conditions. Therefore, Kv7 channel openers/enhancers or drugs acting partly through these channels have been proposed as bronchodilators, expectorants, antitussives, chemotherapeutics and pulmonary vasodilators.

## Lung Diseases: One World’s Health Concern

**Figure 1:**
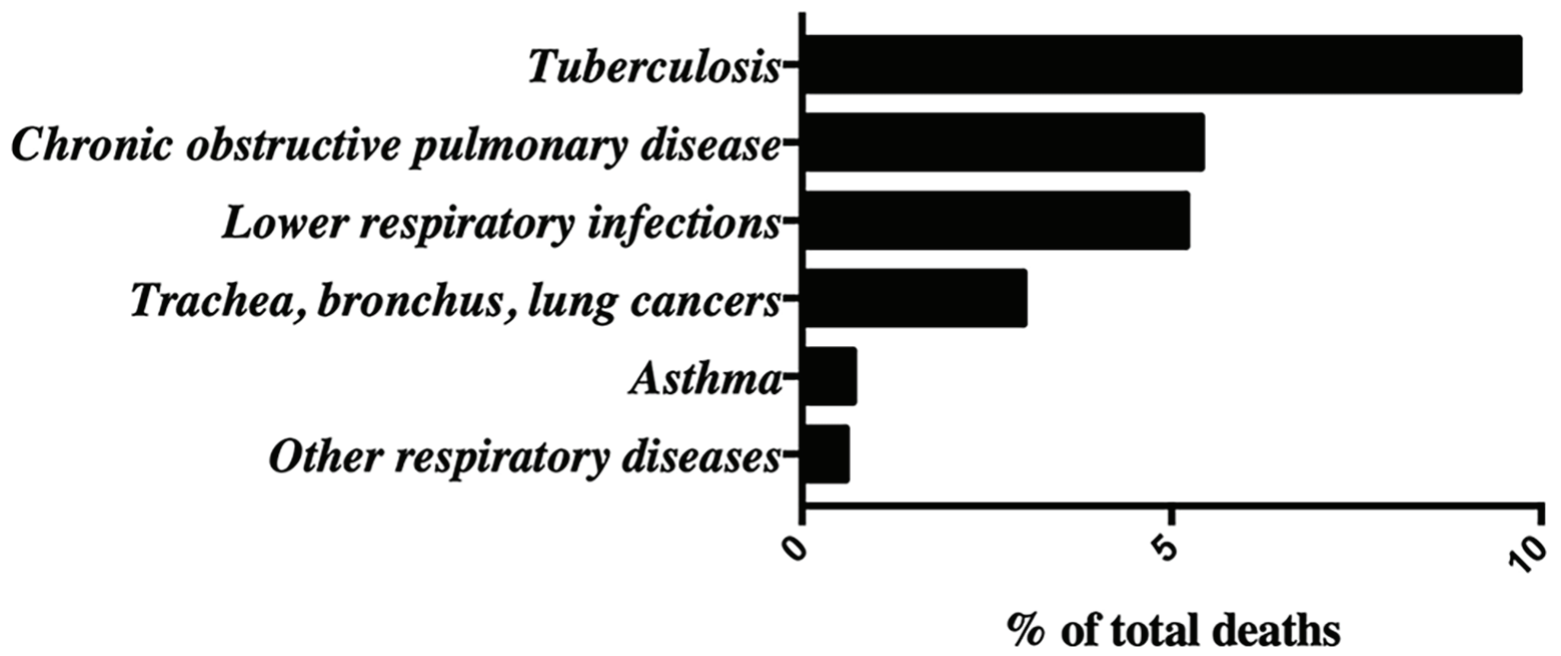
Global lung diseases epidemiological data adapted from the *World Health Organization (WHO) Website*: Mortality rates in 2016 measured as % of total deaths.

Lung diseases constitute a global health concern causing severe physical limitations and premature death, and entails enormous costs associated with treatment, hospital-based and primary care (Tobin, 2005). Mortality from overall lung diseases is very striking according to data collected by the WHO in 2016, in which respiratory diseases account for 24% of world population mortality, the second cause of death after cardiovascular diseases (WHO Global Health Estimates, 2020). At the top of mortality rate within lung diseases, the 9% of the population dies of tuberculosis. Second and third positions include chronic obstructive pulmonary disease (COPD) and lower respiratory infections with 5.4 and 5.2%, respectively. These are followed by lung cancer (affecting trachea, bronchus, and lungs) with 3% and, lastly, asthma and other respiratory diseases with a 0.7 and 0.6%, respectively (Figure 1). Most major respiratory diseases are avoidable and can be mitigated by reducing exposure to air pollution, toxic smoke of biomass fuel, tobacco smoke, and different allergens (Laumbach and Kipen, 2012). Additional factors, such as aging, genetic predisposition, nutritional status, obesity, physical inactivity, raised blood pressure, human immunodeficiency virus 1 infection (HIV-1), or other infections may influence the risk of developing lung diseases. Lung diseases can be classified into three main groups depending on the lung tissue involved. First, airways diseases including asthma, COPD, emphysema, cystic fibrosis, or bronchitis in which the bronchial tubes are narrowed or blocked and the transport of oxygen and other gases are affected (Athanazio, 2012). Second, lung tissue diseases which affect the structure of alveoli or the interstitium (Corvol et al., 2009), such as pneumonia, tuberculosis, pulmonary edema, acute respiratory distress syndrome, sarcoidosis, and pulmonary fibrosis, where the lungs are unable to expand fully (Dellaripa, 2018). The third group includes diseases affecting the pulmonary vessels, such as pulmonary hypertension characterized by vasoconstriction, vascular remodeling, inflammation, and thrombosis (Cummings and Bhalla, 2015). This last group of diseases may end up affecting heart function.

Coronavirus disease 2019 (COVID-19), caused by severe acute respiratory syndrome coronavirus 2 (SARS-CoV-2), has recently rapidly evolved into a global pandemic. To date, risk factors associated with admission to ICU and mortality include cardiovascular comorbidities while respiratory diseases paradoxically do not appear to increase the prevalence or severity of the viral infection (Halpin et al., 2020; Li et al., 2020).

## Kv7 Channel Structure and Regulation

The Kv7 channel family, is a group of voltage-dependent K^+^ channels subfamily Q, which are involved in various physiological activities of numerous cell types, especially, cardiac myocytes, smooth muscle cells, neurons, and epithelial cells (Stott et al., 2014). The different Kv7 channels isoforms are encoded by *KCNQ1–5* genes located at chromosomal loci *11p15*, *20q13*, *8q24*, *1p34*, and *6q13*, respectively (Barrese et al., 2018). *KCNQ* genes encode for five Kv7 proteins, Kv7.1–Kv7.5 channels, comprising approximately 650–940 aminoacids. Each pore-forming α-subunit of Kv7 channels has six helices transmembrane domains (TM). The TM5 and TM6 are part of the pore while the TM4 constitutes the voltage-detection domain. Four α-subunits form the transmembrane pore assembling into a homo‐ or hetero-tetrameric channel (Taylor and Sanders, 2017). Kv7 channels can be regulated by potassium voltage-gated channel subfamily E regulatory subunits (KCNEs) ancillary subunits (KCNE1–5, also called minK-related peptides), which present a single transmembrane domain with 103–177 residues, encoded by the five *KCNE1–5* genes located at chromosomal loci *21q11*, *21q11*, *11q13*, *2q36*, and *Xq23*, respectively (Lundquist et al., 2006). These KCNE subunits modulate several features of Kv7 channels such as biophysical properties, cell localization, channel trafficking, and sensitivity to different drugs (Barrese et al., 2018).

Mutations in all five *KCNQ* and *KCNE* genes have been shown to underlie excitability hereditary disorders (Jentsch, 2000; Maljevic et al., 2010; Lehman et al., 2017; Barrese et al., 2018). Particularly, mutations affecting *KCNQ1* gene cause disorders related to cardiac electrical activity such as long-QT syndrome, short-QT syndrome, and atrial fibrillation leading to severe arrhythmias and sudden death (Li et al., 1998; Chen et al., 2003). *KCNQ2* and *KCNQ3* gene mutations have been identified in different epilepsy syndromes (Neubauer et al., 2008) and, moreover, *KCNQ3* gain-of-function variant have been related to autism and developmental disability (Sands et al., 2019). Kv7.4 channels expressed in the cochlea are essential for normal hearing, and *KCNQ4* mutations are linked to hearing loss (Kubisch et al., 1999; Jung et al., 2019). Lastly, mutations in the Kv7.5 channel encoded by *KCNQ5* gene cause epilepsy and intellectual disability (Lehman et al., 2017). Different mutations have also been found in all five *KCNE* genes, mainly related to cardiac arrhythmias (Splawski et al., 2000; Lundby et al., 2008; Ohno et al., 2009; Abbott, 2016).

Different combinations of Kv7 channels and KCNE subunits can be found depending on the cell type. Thus, there is a Kv7 channel isoform-specific expression playing different roles depending on tissue types (see section Kv7 Channel Expression in the Lung). Many KCNE subunits have been shown to modulate Kv7 channels α-subunits expressed in heterologous systems, providing a valuable experimental platform for elucidating structure-function relationships and mechanistic differences. Thus, all five KCNE proteins have been demonstrated to modulate Kv7.1 channels expressed in heterologous systems with diverse effects (Van Horn et al., 2011; Sun and MacKinnon, 2020). For instance, KCNE1 causes the Kv7.1 channel to activate at more positive voltages, slows its activation and deactivation, increases its conductance, and suppresses its inactivation (Sun and MacKinnon, 2020). While, KCNE2 and KCNE4 have inhibitory effects on Kv7.1 activity (Grunnet et al., 2002; Vanoye et al., 2009; Hu et al., 2019), KCNE3 stabilizes Kv7.1 channels voltage sensors (S4 segment) in an activated state turning the channel voltage-independent (Barro-Soria et al., 2015). Such a mechanism seems to require the participation of the signaling lipid phosphatidylinositol 4,5-bisphosphate (PIP_2_) in non-excitable cells such as lung epithelial cells (Zhou et al., 2019; Sun and MacKinnon, 2020). Sun and MacKinnon have recently identified binding sites of PIP_2_ on Kv7.1 channels present in the S0, the S2-S3 loop, and the S4-S5 linker, and proposed a model in which the conformational change following PIP_2_ interaction would results in dilation of the pore’s gate (Sun and MacKinnon, 2020). In addition, KCNE subunits regulate traffic and cell surface expression of Kv7.1 channels (Roura-Ferrer et al., 2010; Hu et al., 2019). The information about the regulation of Kv7.2 and Kv7.3 channels by KCNE subunits is more limited. In *Xenopus* oocytes, KCNE1 was found to slow Kv7.2/7.3 heteromer currents and decreased their magnitude (Yang et al., 1998). Likewise, KCNE2 was shown to accelerate the deactivation kinetics of Kv7.2 and Kv7.2/7.3 channels transiently expressed in COS cells (Tinel et al., 2000). Whereas, KCNE1, KCNE2, and especially KCNE4 and KCNE5 increased Kv7.4 currents, co-expression with KCNE3 led to a strong attenuation (Strutz-Seebohm et al., 2006; de Jong and Jepps, 2018). Moreover, KCNE4 was shown to co-localize with Kv7.4 channels, regulating its activity and membrane abundance in mesenteric arterial myocytes (Jepps et al., 2015). Finally, Kv7.5 channels were shown to be affected by KCNE1 and KCNE3, but not by other KCNE subunits in *Xenopus* oocytes and HEK-293 cells (Roura-Ferrer et al., 2009, 2012). Thus, while KCNE1 was shown to slow activation and increase Kv7.5 currents, KCNE3 drastically inhibited these currents (Roura-Ferrer et al., 2009, 2012).

The activity of Kv7 channels is modulated by G protein-coupled membrane receptors (GPCRs). Thus, activation of different GPCR have been shown to inhibit M-type (Kv7) currents, with a dominant participation of the G_q_ and G_11_ α-subunits (Caulfield et al., 1994; Haley et al., 1998; Delmas and Brown, 2005). For instance, in neurons, Kv7 activity is decreased by M_1_ and M_3_ muscarinic and H_1_ histaminic receptors coupled to G_q/11_ proteins through different signal transduction pathways, including depletion of PIP_2_, activation of protein kinase C (PKC), or increased intracellular calcium (Delmas and Brown, 2005; Higashida et al., 2005; Taylor and Sanders, 2017; Barrese et al., 2018). In human airway smooth muscle cells, histamine was found to inhibit Kv7.5 channels though a PKC-dependent phosphorylation of Ser441 (Haick et al., 2017). On the other hand, it is well recognized that stimulation of β-adrenergic receptor increases the activity of different Kv7 channels in different tissues such as cardiac (Kv7.1), neuronal (Kv7.2/Kv7.3), and vascular (Kv7.4/Kv7.5 and Kv7.5) channels (Jentsch, 2000; Marx et al., 2002; Chadha et al., 2014; Mani et al., 2016; Barrese et al., 2018). Likewise, in human airway smooth muscle cells, Kv7.5 channels are robustly enhanced by activation of β-adrenoceptors *via* a mechanism involving cyclic adenosine monophosphate/protein kinase A (cAMP/PKA)-dependent phosphorylation (Brueggemann et al., 2018). This effect might be shared with other GPCRs but does not seem to account for Kv7.4 activation. The mechanism may involve increased membrane expression of Kv7 channels mediated by colchicine-sensitive microtubule disruption (Lindman et al., 2018). Kv7 channels are also regulated by GPCR-independent mediators activating soluble guanylate cyclase (sGC) such as nitric oxide (NO) (Mondejar-Parreño et al., 2019) and natriuretic peptide (Stott et al., 2015). The implications in respiratory diseases and drug therapy are discussed below.

In addition to regulation by KCNE β-subunits and GPCRs, Kv7 channels can be regulated by scaffolding proteins, sodium-coupled myo-inositol transporter (SMIT), lipids, microRNAs, and/or further signaling mechanisms as extensively reviewed elsewhere (Delmas and Brown, 2005; Zaydman and Cui, 2014; Taylor and Sanders, 2017; Barrese et al., 2018; Byron and Brueggemann, 2018).

## Kv7 Channel Expression in the Lung

**Figure 2:**
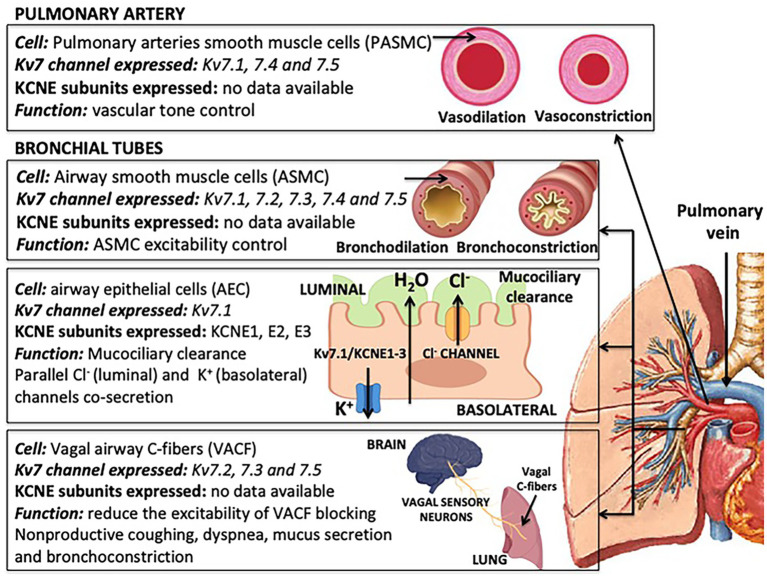
Expression and functions of isoform-specific Kv7 channel and potassium voltage-gated channel subfamily E regulatory subunits (KCNE) ancillary subunit in lung cells.

Kv7 channels are expressed mainly in pulmonary arteries (PAs) and in the bronchial tubes (Figure 2). In PA, Kv7 channels are expressed in pulmonary artery smooth muscle cells (PASMCs); while in the bronchial tubes (trachea, bronchus and bronchioles), Kv7 channels are expressed in airway smooth muscle cells (ASMCs), airway epithelial cells (AEPs) and vagal airway C-fibers (VACFs). The functional role of Kv7 channels may vary depending on the cell type. While most of the available studies have provided relevant information on the particular expression of α-subunits in these cell types, evidence for the expression of heteromeric Kv7 complexes or KCNE subunits is still very scarce.

### Pulmonary Artery Smooth Muscle Cells

The expression of Kv7 channels in mice and rat PA has been demonstrated in several studies. Specifically, their expression is located in PASMC. In these cells, the expression of Kv7.1, Kv7.4, and Kv7.5 channels predominates; but Kv7.4 channels expression appears to be the most relevant. The expression of Kv7.2 and Kv7.3 channels in PASMCs is still controversial (Joshi et al., 2009; Morecroft et al., 2009; Chadha et al., 2012; Li et al., 2014a; Sedivy et al., 2015; Eid and Gurney, 2018; Mondejar-Parreño et al., 2018). The existence of Kv7.1/7.5 or Kv7.4/7.5 heteromeric complexes in vascular smooth muscle has been postulated on several studies (Chadha et al., 2014; Oliveras et al., 2014; Barrese et al., 2018). In particular, Kv7.4/7.5 heteromers have been proposed as the dominant functional vascular Kv7 channels, supported by electrophysiological data showing that Kv7 currents in freshly isolated vascular smooth muscle cells have phenotypes intermediate to the respective homomeric Kv7.4 and Kv7.5 channels (Brueggemann et al., 2014a; Chadha et al., 2014; Fosmo and Skraastad, 2017; Barrese et al., 2018). We have recently confirmed that Kv7.4/7.5 heteromeric complexes are also present in PASMC (authors’ unpublished observations). Although, in other types of arteries, the expression of Kv7 channels has been located also in endothelial cells (Goodwill et al., 2016), whether this is also the case for pulmonary artery endothelial cells (PAECs) remains unknown. The main function of these channels is to regulate pulmonary vascular tone, due to their contribution to maintain the membrane potential (Em) of PASMC (Joshi et al., 2009; Sedivy et al., 2015; Mondejar-Parreño et al., 2018, 2019).

### Airway Smooth Muscle Cells

There is some interspecies variability in the expression of Kv7 channel isoforms in the airways. Thus, ASMCs from guinea pigs were shown to express Kv7 channels with a relative abundance of *KCNQ2* > *KCNQ5* > *KCNQ4* > *KCNQ3* and, to a lesser extent, *KCNQ1* (Brueggemann et al., 2011). Comparatively, *KCNQ* mRNA expression in ASMC revealed a high expression of *KCNQ4* followed by a fainter expression of *KCNQ5* and *KCNQ1* transcripts (Brueggemann et al., 2014b). Studies in human tracheal muscle revealed abundant expression of *KCNQ1* relative to guinea pig, a modest expression of *KCNQ4* and *KCNQ5* and no detectable expression of *KCNQ2* or *KCNQ3* (Brueggemann et al., 2011). A further study showed that cultured human ASMCs express all *KCNQ* genes with a predominant relative abundance of *KCNQ5* mRNA followed by *KCNQ3* or *KCNQ4*, while *KCNQ2* and *KCNQ1* mRNA are expressed to a lesser extent (Brueggemann et al., 2018). These differences in the expression profile of Kv7 channels in human tissue could be due to phenotypic changes associated with cell culture. Measurements of Kv7 currents and functional studies assessing airway smooth muscle contractility in the bronchioles from different species have pointed to Kv7 channels as important players in maintaining, resting Em, and controlling ASMC excitability (Evseev et al., 2013; Brueggemann et al., 2014b, 2018).

### Airway Epithelial Cells

Airway epithelial cells (AECs) predominates the expression of Kv7.1 channels, also named KvLQT1, along with the expression of KCNE1 and KCNE3 ancillary subunits (Mall et al., 2000; Boucherot et al., 2001; Trinh et al., 2007; Preston et al., 2010; Frizzell and Hanrahan, 2012; Kroncke et al., 2016; Hynes and Harvey, 2019). Nevertheless, KCNE2 has also been found to be expressed in *Calu-3* cells, a model for human submucosal serous cells of the lung, where it has been suggested to regulate *KCNQ1* (Cowley and Linsdell, 2002a). Chloride (Cl^−^) secretion in AEC is essential to mucociliary clearance and requires the co-activation of Cl^−^ channels in luminal membrane and, in parallel, of K^+^ channels in the basolateral membrane which involves Kv7.1 channels (Frizzell and Hanrahan, 2012).

### Vagal Airway C-Fibers

Using single-cell mRNA, Sun et al. have recently demonstrated that *KCNQ2*, *KCNQ3*, and *KCNQ5* are consistently expressed in mouse airway sensory afferent nerves and remarkably, the expression of *KCNQ3* mRNA was the most prevalent (Sun et al., 2019). Most vagal sensory nerves in the respiratory tract are nociceptive C-fibers. Increased VACF activity may contribute to the nonproductive coughing, dyspnea, parasympathetic reflex mucus secretion, and bronchoconstriction. Thus, it has been proposed that activation of Kv7 channels may reduce the vagal C-fibers excitability innervating the airways, and being useful to treat chronic cough.

## Kv7 Channels in Lung Diseases

This section covers the potential pathophysiological role of Kv7 channels in different respiratory diseases such as cystic fibrosis, asthma, COPD, chronic coughing, lung cancer, and pulmonary hypertension.

### Cystic Fibrosis

The airway surface liquid layer represents a key primary defense mechanism of mucociliary clearance, which crucially depends on the transport of chloride into the lumen of the airways, and the subsequent movement of water. Cl^−^ ion transport across the apical membrane is mainly driven by a cAMP-regulated Cl^−^ channel named cystic fibrosis transmembrane conductance regulator (CFTR) and by the calcium-activated Cl^−^ channels (CaCC) (Frizzell and Hanrahan, 2012; Bartoszewski et al., 2017). However, a possible overlap between these two pathways has also been proposed (Billet and Hanrahan, 2013). Cystic fibrosis is an autosomal recessive disease due to mutations in the gene encoding CFTR and characterized by alterations in the composition and volume of secreted luminal fluids. The CFTR is a member of the ATP-binding cassette (ABC) transporter superfamily known to function as an ion cannel that conducts chloride ions across epithelial cell membranes and is responsible for salt secretion in response to cAMP/PKA stimulation. Thus, mutations of the CFTR gene lead to defective cAMP-dependent Cl^−^ secretion and enhanced Na^+^ absorption throughout the apical membrane of airway epithelia.

CFTR has been shown to influence both function and properties of other membrane channels, such as epithelial Na^+^ channels, inward rectifying renal outer medullary K^+^ channel (ROMK), outwardly rectifying Cl^−^ channels, and aquaporin 3 (Schwiebert et al., 1999). Cl^−^ secretion in AEC requires the parallel co-activation of Cl^−^ channels and K^+^ channels, in the luminal surface and the basolateral membrane, respectively. Whereas Cl^−^ channels are required for Cl^−^ efflux to the luminal side of the AEC, K^+^ channels are needed for K^+^ recycling *via* the basolateral membrane (Boucherot et al., 2001; Bartoszewski et al., 2017). Kv7.1 channels have been shown to contribute to the basolateral cAMP-activated K^+^ conductance in human airway epithelium (Mall et al., 1996, 2000; Bardou et al., 2009). This study also showed that the Kv7.1 channel inhibitor chromanol 293B inhibited Cl^−^ secretion suggesting an important role of these K^+^ channels in maintaining cAMP-dependent Cl^−^ secretion in human airways. Since CFTR is a modulator of other ion channels, several studies have analyzed whether it controls the activity of Kv7.1 channels. Studies analyzing this issue have led to contradictory results. Thus, while initial studies suggested that CFTR could directly modulate Kv7.1 conductance (Cunningham et al., 1992; Loussouarn et al., 1996; Mall et al., 2000), this was not the case in other studies (Boucherot et al., 2001). Interestingly, using CFTR KO mice, Belfodil et al. (2003) found that cAMP-sensitive Kv7.1 currents were regulated by CFTR in renal distal but not in proximal tubule cells, suggesting that the possible modulation of Kv7.1 is unlikely to involve a direct interaction.

As stated above, human airway epithelia also express CaCC that are activated by extracellular nucleotides [adenosine triphosphate (ATP) and/or uracil triphosphate (UTP)] and other Ca^2+^-increasing agonists. CaCC was shown to be preserved or enhanced in human and murine cystic fibrosis airways as a mechanism to compensate for the lack of CFTR (Clarke et al., 1994; Grubb et al., 1994). Since, CaCC-mediated Cl^−^ secretion appears to be preserved in cystic fibrosis its pharmacological stimulation has been suggested as a therapeutic tool for bypassing dysfunctional CFTR. Mall et al. demonstrated that activation of the cAMP pathway increased CaCC-mediated secretion in cystic fibrosis and, conversely, chromanol 293B, an inhibitor of cAMP-activated Kv7.1 channels was able to fully abolish nucleotide-activated Cl^−^ secretion (Mall et al., 2003). Hence, the authors proposed that activation of Kv7.1 channels, together with hSK4 K^+^ channels, may improve Cl^−^ secretion *via* CaCC in human cystic fibrosis airway tissues (Mall et al., 2003). Altogether, these studies indicate the Kv7.1 channel activation may help to improve mucociliary clearance in cystic fibrosis airways.

Elevated levels of reactive oxygen species (ROS) lead to oxidative stress and DNA damage in several tissues providing a common pathological mechanism in all inflammatory lung diseases. Hydrogen peroxide (H_2_O_2_) has been proven to stimulate several components of the Cl^−^ secretion in the airway epithelia, including the cAMP-dependent activation of CFTR and the basolateral K^+^ channels (Cowley and Linsdell, 2002b; Ivonnet et al., 2015). The basolateral K^+^ conductance was abrogated by clofilium, a Kv7.1 channel inhibitor, suggesting a role for these channels in the H_2_O_2_-stimulated response (Cowley and Linsdell, 2002b; Ivonnet et al., 2015). In line with this, H_2_O_2_ has been reported to potently enhance Kv7 currents (Gamper et al., 2006; Kim et al., 2016). Activation of transmembrane adenylyl cyclase by H_2_O_2_ has been proposed as a triggering mechanism for the cAMP-activated basolateral Kv7.1 current (Ivonnet et al., 2015). The stimulated anion secretion by ROS could act as a compensatory protective mechanism to keep the airways clear from damaging radicals and to hold acceptable airway surface liquid levels.

While the Kv7.1 channel is the member of the *KCNQ* family with the strongest evidence of its functional expression and physiological role in lung epithelial cells, others (*KCNQ3* and *KCNQ5*) have been detected in the apical or lateral membranes (Moser et al., 2008). In addition, mRNA transcripts for several KCNE accessory subunits (*KCNE2* and *KCNE3*) have been found in human airway Calu-3 cells (Cowley and Linsdell, 2002a). Kv7.1 channels expressed in *Xenopus* oocytes were shown to be activated by an increase in intracellular cAMP concentration, independently of co-expression with KCNE1 or KCNE3 ancillary subunits (Boucherot et al., 2001). On the other hand, Potet et al. demonstrated that recombinant Kv7.1/KCNE1 complexes expressed in *Cos-7* cells were totally insensitive to cAMP (Potet et al., 2001). The co-assembly of Kv7.1 with KCNE3 subunits markedly affects Kv7.1 channel properties (abolishing its voltage dependence) and has been proposed to form the cAMP-activated basolateral K^+^ channels contributing to Cl^−^ secretion in colonic epithelial cells (Schroeder et al., 2000). Interestingly, Preston et al. evidenced that cAMP-stimulated Cl^−^ secretion across tracheal epithelia was severely reduced in *KCNE3*^(−/−)^ mice (Preston et al., 2010). While the involvement of KCNE3 in airway secretion requires confirmation in future studies, the structural basis of the interaction of Kv7.1 with KCNE3 leading to a constitutive activation of Kv7.1 channel activity has been recently established (Kroncke et al., 2016; Sun and MacKinnon, 2020).

### Asthma

Asthma is characterized by hyper-contractility of bronchial smooth muscle attributed to an increased concentration of different bronchoconstrictor agonists, or enhanced sensitivity to them, leading to aberrant narrowing of the airways (Penn, 2008). Acetylcholine, leukotriene D4, endothelin, and histamine represent the most relevant locally released bronchoconstrictor agonists that activate GPCR on ASMC, leading to increased cytosolic Ca^2+^ concentration and contraction (Jude et al., 2008; Penn, 2008). This together with the strong inflammation leads to an acute hypersensitivity to different air stimulants that produce ASMC bronchospasm and that, in the long term, promote smooth muscle proliferation and hypertrophy (Stott et al., 2014). Kv7 channels are considered negative regulators of ASMC contraction due to their major contribution to maintain negative Em negative, preventing L-type-Ca^2+^ channel activation and the subsequent bronchoconstriction (Brueggemann et al., 2011). Several members of the Kv7 family are expressed in ASMCs (Brueggemann et al., 2011; Bartoszewski et al., 2017). The relative abundance expression of the Kv7 members appears to differ depending on the location (trachea vs. bronchioles) and depending on the animal species as mentioned above (Brueggemann et al., 2011, 2014b Evseev et al., 2013). Several studies have evidenced that bronchoconstrictor agonists negatively regulate the activity of Kv7 channels. Thus, methacholine, carbachol, or histamine reduce Kv7 currents in guinea pig, mouse, and rat ASMCs (Brueggemann et al., 2011, 2014; Haick et al., 2017). On the other hand, structurally unrelated Kv7 channel activators cause a concentration-dependent relaxation of small bronchioles (Brueggemann et al., 2014b) and tracheal rings (Evseev et al., 2013). Of note, *in vivo* administration of the Kv7 channel opener retigabine was proven to transiently reduce bronchoconstriction induced by methacholine in mice (Evseev et al., 2013).

Chronic administration of long-acting β_2_-adrenergic receptor agonist elicits a time‐ and concentration-dependent relaxation of rat airways, with remarkable desensitization to short-term or with repeated treatments. Co-administration of retigabine plus the long-acting β_2_-adrenergic receptor agonist formoterol was shown to cause a sustained reduction of methacholine-induced bronchoconstriction and reduced the desensitization observed with formoterol alone (Brueggemann et al., 2014b). These findings suggest that using a Kv7 channel enhancer together a β_2_-adrenergic receptor agonist may improve bronchodilator therapy.

In addition to exaggerated bronchoconstriction, asthma is characterized by a chronic inflammatory response in the airways that is targeted primarily with inhaled corticosteroids. As stated in the previous section basolateral membrane K^+^ channels play a key role in stimulating Cl^−^ transport across AEPs (Frizzell and Hanrahan, 2012). Unlike in cystic fibrosis, a decrease in airway mucus secretion ameliorates the symptomatology of asthma. Very recently, Hynes and Harvey have demonstrated that dexamethasone produces an inhibition of transepithelial chloride ion secretion though a rapid non-genomic inhibition of Kv7.1 channels and KCNN4 (K_Ca_3.1) (Hynes and Harvey, 2019). Thus, it is plausible that down-regulation of Kv7.1 channel activity might contribute to the potential benefit of corticosteroids to reverse airway hypersecretion.

### Chronic Obstructive Pulmonary Disease (COPD)

COPD is a progressive life-threatening lung disease characterized by airway obstruction due to inflammation of the small airways. The disease evolves with irreversible limitation of airflow, loss of lung function and severe acute exacerbations which significantly deteriorate the patient’s quality of life (Barnes et al., 2003). β-adrenergic receptors (β-AR) agonists promote airway smooth muscle relaxation and are the drugs of choice for rescue from acute bronchoconstriction in asthma and COPD. A recent study has shown that the long-acting β-AR formoterol robustly enhanced Kv7 currents in human ASMCs (Brueggemann et al., 2018). Furthermore, these authors provided evidence that Kv7.5 is the predominant Kv7 α-subunit in human ASMCs and identified a PKA-dependent phosphorylation of S53 on the N-terminus of Kv7.5 channel subunits as a potential mechanism of activation following the stimulation of the βAR/cAMP pathway (Brueggemann et al., 2018).

The exposure to cigarette smoking is the principal cause of COPD (Salvi, 2014). Recently Sevilla-Montero et al. have analyzed the impact of cigarette smoke exposure on the pulmonary vasculature (Sevilla-Montero et al., 2019). Authors showed that cigarette smoke extract exposure had direct effects on human fibroblasts and PASMCs, promoting a senescent phenotype which contributed to the secretion of inflammatory molecules and increase the proliferation of non-exposed cells. Furthermore, cigarette smoke extract exposure affected cell contractility attenuated the pulmonary vascular responses to Kv7 channel modulators and dysregulated the expression and activity of Kv7.4 channels. The results suggest that cigarette smoke may impair pulmonary vascular tone atleast in part through Kv7.4 downregulation (Sevilla-Montero et al., 2019).

A sequence polymorphism of the human *KCNE2* gene has been found to be associated with altered lung function (Soler-Artigas et al., 2011; Sabater-Lleal et al., 2014). The deletion of *KCNE2* reduced pulmonary expression of the Kv7.1 α-subunit suggesting the formation of pulmonary KCNQ1-KCNE2 complexes (Zhou et al., 2019). *KCNE2*^(-/-)^ mice had reduced blood O_2_, increased CO_2_, increased pulmonary apoptosis, and increased inflammatory mediators TNF-α, IL-6, and leukocytes in bronchoalveolar lavage fluids (Zhou et al., 2019). These data indicated KCNE2 regulates *KCNQ1* in the lungs and is required for normal lung function.

### Chronic Cough

Airway sensory afferent nerves are essential for triggering different reflex responses to potential injurious stimuli (Undem and Kollarik, 2005). Most vagal sensory afferent nerves which terminate in the respiratory tract are C-fibers (Undem and Kollarik, 2005). The excessive activity of VACF induces different symptoms associated with inflammatory lung diseases, such as nonproductive coughing, dyspnea, parasympathetic reflex mucus secretion and bronchoconstriction (Mazzone and Undem, 2016). Consequently, ion channels that regulate the excitability of C-fibers in the airways can be an attractive therapeutic target aimed at reducing the symptoms of various inflammatory diseases of the airways. Using single-cell mRNA analysis, Sun and collaborators have very recently shown that *KCNQ2*, *KCNQ3*, and *KCNQ5* are consistently expressed in mouse lung specific nodose neurons with *KCNQ3* being the most predominant (Sun et al., 2019). This study suggested that Kv7.3 channels are the major contributors to the Kv7-M current in the nodal neurons of the mouse lungs. The Kv7 channel activator retigabine increased the amplitude and accelerated the activation rate of the M-current, caused a prominent hyperpolarization, and suppressed the excitability of mouse nodose neurons. These effects were reversed or prevented in the presence of XE991, a Kv7 channel inhibitor. Remarkably, nebulized retigabine was found to attenuate cough evoked by inhalation of irritant gases (SO_2_ and NH_3_) in awake mice (Sun et al., 2019). Although future studies are needed to confirm their role in the regulation of C-fiber excitability of the airways in humans, these data indicate that Kv7.3 channels may provide a novel therapeutic target to reduce the conditions associated with pulmonary inflammatory diseases such as chronic coughing.

### Non-small Cell Lung Cancer

K^+^ channels regulate a number of processes of key relevance in cancer biology such as cell volume control, pH regulation, migration, proliferation, differentiation, apoptosis, and drug resistance (Rao et al., 2015; Prevarskaya et al., 2018; Haworth and Brackenbury, 2019; Serrano-Novillo et al., 2019). Thus, there is increasing evidence that K^+^ channels play an important role in cancer; but a clear scenario is still far due to the plethora of K^+^ channel isoforms and their diverse repertoire of effects. Notably, K^+^ channels regulate cell cycle, but according to the channel isoform, its level of expression, the cell type, and the stage of differentiation the effects are diverse. For instance, Kv channels may exhibit proliferative or anti-proliferative roles, depending on the channel subtype and the phase of the cell cycle (Rao et al., 2015). Moreover changes in the phenotypes, signaling pathways, and pattern of expression of K^+^ channels may be observed in cancer, which increases the complexity of K^+^ channels regulation on cell viability. In addition to the well-characterized involvement of several Kv channels (most notably Kv1.1, Kv1.3, Kv1.5, Kv10.1, Kv10.2, and Kv11.1) in cancer (Rao et al., 2015), some members of the Kv7 family have also been suggested to participate in cell proliferation and cancer (Serrano-Novillo et al., 2019).

K^+^ channels participate in tumor cell invasion and metastasis propagation *via* regulation of cell migration and growth (O’Grady and Lee, 2005; Schwab et al., 2008; Arcangeli et al., 2009; Prevarskaya et al., 2010). Indeed, inhibiting or silencing K^+^ channels reduces the proliferation of cancer cells (Pancrazio et al., 1993; Jang et al., 2011). Kv7.1 channels have been highlighted in cell migration, proliferation, and repair of AECs (Trinh et al., 2007). In 2014, Girault and colleagues showed an increased expression (by 1.5‐ to 7-fold) of Kv7.1 channels in tumor lung adenocarcinoma samples, compared to non-neoplastic tissues from 17 of 26 patients (Girault et al., 2014). Authors found that the proliferation rates of lung adenocarcinoma cell monolayers were reduced by the Kv7.1 channel blockers clofilium and chromanol, or after silencing Kv7.1 channels using a specific siRNA. These data suggest that Kv7.1 channels could be a potential therapeutic target in lung cancer (Girault et al., 2014).

On the other hand, K^+^ channels have also been implicated in both early and late stages of apoptosis. The decrease in cell volume is one of the earliest and necessary morphological changes observed in cells undergoing apoptosis. Active K^+^ channels in the plasma membrane leads to K^+^ and Cl^−^ outflow through K^+^ and Cl^−^ channels, this generates an osmotic gradient leading to an outward water transport through aquaporins which results in a decrease in cell volume (Maeno et al., 2000). In later stages of apoptosis, the augmented intracellular K^+^ concentration ([K^+^]_int_) suppresses caspase and nuclease activity and, conversely, the decreased [K^+^]_int_ caused by K^+^ channel activation, promotes apoptosis (Burg et al., 2008). There is a wealth of literature supporting that the M‐ channel activators have a neuroprotective role *via* Kv7 activation and reduction of hyperactivity and, hence, suppression of glutamate excitotoxicity (Gamper et al., 2006; Bierbower et al., 2015; Vigil et al., 2020). It is likely that flupirtine would work the same way (Panchanathan et al., 2013; Li et al., 2014a). Nevertherless, its ability to inhibit cell viability has also been reported. Thus, Lee et al. showed that flupirtine exerted anti-proliferative effects in canine osteosarcoma cells by enhancing Kv7.5 function and arresting cells in the G_0_/G_1_ phases (Lee et al., 2014). Similarly, retigabine was found to exhibit anti-proliferative effects in *C2C12* myoblasts (Iannotti et al., 2010).

Non-small-cell lung cancer (NSCLC) accounts for most cancer deaths. Aberrant activation of the epidermal growth factor receptor (EGFR) is involved in the pathogenesis and progression of several human cancers, including NSCLC (Melosky, 2014). Indeed, approximately 10–38% of NSCLC patients have EGFR gene mutations (Lin et al., 2014). EGFR tyrosine kinase inhibitors, such as Gefitinib have become standard first-line treatment for these patients (Cohen et al., 2003). However, acquired resistance represents a major limitation for drug efficacy. Cancer stem cells have self-renewal properties in various solid tumors, and play a key role in tumor growth and progression (Wang et al., 2013) and in therapy resistance (Steinbichler et al., 2018). A subset of cancer stem cells, termed the “side population” (SP), have elevated clonogenic potential and higher expression levels of ABC-transporters than main-population cells known as non-SP cells (Richard et al., 2013) and are considered to be responsible for anti-cancer drug resistance (Singh et al., 2010; Leon et al., 2016). Choi and colleagues have demonstrated a reduced mRNA expression of *KCNQ3* and *KCNQ5* in SP cells compared to non-SP cells (Choi et al., 2017). The treatment of flupirtine (a Kv7.2–7.5 channel enhancer) decreased the viability of SP and non-SP cells. In addition, the combination treatment of gefitinib plus flupirtine was more effective, compared to the individual treatment of both drugs, in decreasing the viability of both non-SP cells, and gefitinib-resistant SP cells. According to their results, the combination treatment of gefitinib plus flupirtine was proposed to reduce the viability of gefitinib-resistant cells through inhibition of the EGFR-Ras-Raf-ERK pathway *via* (Choi et al., 2017). These results suggest that the activation of Kv7.3 and Kv7.5 channels could play an important role in the induction of apoptosis in SP cells, either by participating in the decrease of the cellular volume necessary for apoptosis initiation; or by decreasing [K^+^]_int_, which results in the activation of caspases and nucleases.

### Pulmonary Hypertension

Pulmonary hypertension is defined by an increase in mean pulmonary arterial pressure at rest, as assessed by right heart catheterization. Based on pathophysiological findings, clinical presentation, hemodynamic characteristics, and therapeutic considerations, pulmonary hypertension is categorized into five groups by WHO: pulmonary arterial hypertension (PAH), pulmonary hypertension due to left-sided heart disease, pulmonary hypertension due to lung disease or hypoxia, chronic thromboembolic pulmonary hypertension, and multifactorial pulmonary hypertension (Simonneau et al., 2019). PAH is characterized by excessive pulmonary vasoconstriction and progressive remodeling of the distal PAs, resulting in elevated pulmonary vascular resistance and, eventually, in right ventricular failure (McLaughlin et al., 2015). Reduced expression or function of K^+^ channels (notably Kv1.5 and TASK-1 channels) in PASMCs results in a more depolarized Em, and is considered to contribute to the enhanced vasoconstriction and proliferation (Moudgil et al., 2006; Boucherat et al., 2015; Hayabuchi, 2017). In 2009, Joshi et al. demonstrated that Kv7 channel blockers, linopirdine, and XE991, inhibited the I_KN_ current in PASMCs and raised mean pulmonary arterial pressure (mPAP); while the Kv7 channel openers, retigabine and flupirtine, had the opposite effects (Joshi et al., 2009). Linopirdine can also increase mesenteric vascular resistance and systemic arterial pressure (Mackie et al., 2008). Functional expression of Kv7 channels in the pulmonary circulation was later confirmed by others (Morecroft et al., 2009; Eid and Gurney, 2018; Mondejar-Parreño et al., 2018). As in the systemic circulation, *KCNQ1* and especially *KCNQ4* and *KCNQ5* appear the most predominant *KCNQ* channels in the pulmonary circulation (Joshi et al., 2009; Morales-Cano et al., 2014; Sedivy et al., 2015; Mondejar-Parreño et al., 2018). The lack of selective drugs for the different Kv7 subunits hinders identification of the functional impact of individual Kv7 members (Barrese et al., 2018). Structurally related Kv7 enhancers such as retigabine and flupirtine (which activate all Kv7 isoforms except Kv7.1 channels) (Barrese et al., 2018) and zinc pyrithione (which activate all Kv7 members but Kv7.3) have been shown to induce pulmonary vasodilation (Joshi et al., 2009; Morecroft et al., 2009; Sedivy et al., 2015; Eid and Gurney, 2018; Mondejar-Parreño et al., 2018). In addition, drugs that block all Kv7 channels (i.e., linopirdine and XE991) are able to contract PAs previously primed (Chadha et al., 2012; Sedivy et al., 2015) or not (Joshi et al., 2009), whereas selective Kv7.1 blockers HMR1556, L768, L673, and JNJ39490282 (Barrese et al., 2018) have no contractile effects in PAs (Chadha et al., 2012). On the other hand, the application of R-L3, a compound purported to activate Kv7.1 channels, relaxed efficiently precontracted intrapulmonary arteries (Chadha et al., 2012; Mondejar-Parreño et al., 2018). These data suggest that even when Kv7.4 and Kv7.5 appear to be the most relevant Kv7 channels in controlling resting Em in PASMC, Kv7.1 channels are also functionally expressed.

The NO/cGMP pathway represents a key physiological signaling controlling tone in PAs, and drugs stimulating this pathway are used to treat PAH (Barnes and Liu, 1995), and drugs activating this pathway are used to treat pulmonary arterial hypertension. Recently, we have found that NO donors and riociguat, a stimulator of sGC used for the treatment of pulmonary arterial hypertension, enhance Kv7 current and induce membrane hyperpolarization in fresh isolated PASMCs contributing to pulmonary vasodilation (Mondejar-Parreño et al., 2019). Further, we identified Kv7.5 channels as a potential member of the Kv7 family targeted by these drugs.

The expression of Kv7 channels has been explored in several experimental models of pulmonary hypertension. While the hypoxic pulmonary vasoconstriction (HPV) reflects a physiological intrinsic property of PASMCs, which allows shifting blood flow from hypoxic to normoxic lung areas, coupling ventilation and perfusion, the chronic exposure to hypoxic air causes the pulmonary circulation to become hypertensive (Sylvester et al., 2012). Sedivy et al. found a significant loss of *KCNQ4* mRNA expression in PA from rats maintained for 3 days in a hypoxic environment, even when a significant reduction of the Kv7.4 protein expression could not be confirmed (Sedivy et al., 2015). On the other hand, the expression of *KCNQ1* and *KCNQ5* was unaffected. However, Li et al. reported that longer incubation on hypoxia (9 days) was associated with a downregulation of *KCNQ5* in the PAs (Li et al., 2014b). These authors also demonstrated that microRNA-190 (miR-190), whose expression is increased following the hypoxic exposure, directly targeted *KCNQ5*. This led the authors to propose the increased in miR-190 as a mechanism involved in the *KCNQ5* downregulation and enhanced vasoconstriction induced by hypoxia. In addition to the hypoxic pulmonary hypertension models, a slight reduction of *KCNQ5* expression has also been observed in rat lungs from monocrotaline-induced pulmonary hypertension (Morales-Cano et al., 2014). On the other hand, we found the expression of *KCNQ1*, *KCNQ4*, and *KCNQ5* mRNAs unaltered in lungs from Zucker rats, a model of type 2 diabetes, developing increased pulmonary arterial pressure, and right ventricular hypertrophy (Morales-Cano et al., 2019).

The potential impairment of *KCNQ* channels has been also explored in conditions associated with pulmonary hypertension. The HIV-1 infection is an established risk factor for PAH, although the pathological mechanisms underlying the development of HIV-related PAH remain unclear (Butrous, 2015). In a recent study using HIV-transgenic mice expressing seven of the nine HIV viral proteins and wild-type mice we observed that HIV mice had reduced lung expression of *KCNQ1* and *KCNQ5* channels (Mondejar-Parreño et al., 2018). By using vascular reactivity and patch-clamp experimental approaches we found decreased responses to the Kv7.1 channel activator L-364,373 but unaltered responses to retigabine (Mondejar-Parreño et al., 2018). These results suggest that Kv7 channels (especially Kv7.1) are impaired in mice expressing HIV-1 proteins.

Congenital diaphragmatic hernia is a rare congenital anomaly commonly associated with persistent pulmonary hypertension. A recent study showed that the expression of *KCNQ5*, but not that of *KCNQ1* or *KCNQ4*, was significantly downregulated at the mRNA and protein levels in rat lungs from nitrofen-induced congenital diaphragmatic hernia (Zimmer et al., 2017). Likewise, a markedly diminished *KCNQ5* expression was found in the pulmonary vasculature of congenital diaphragmatic hernia fetuses. Since Kv7.5 channels have shown to contribute to NO donors-induced pulmonary vasodilation (Mondejar-Parreño et al., 2019), its reduced availability in congenital diaphragmatic hernia could partly explain the poor therapeutic response to inhaled NO in these patients (Group (NINOS), 1997; Putnam et al., 2016).

Studies in experimental models have shown evidence that drugs activating Kv7 channels may be of benefit in the treatment of pulmonary hypertension with different etiologies (Morecroft et al., 2009; Sedivy et al., 2015). Morecroft et al. also demonstrated that flupirtine attenuated the development of chronic hypoxia-induced pulmonary hypertension in mice and reversed established pulmonary hypertension in mice that over-express the 5-HT transporter (SERT), apparently *via* Kv7 activation (Morecroft et al., 2009). In this study authors showed that the chronic treatment with flupirtine reduced mean right ventricle pressure (mRVP), pulmonary remodeling, and RV hypertrophy (RVH). More recently, Sedivy et al. confirmed the potential benefit of Kv7 channel openers showing the pulmonary antihypertensive effects of flupirtine in rats exposed to short-term (3–5 days) hypoxia (Sedivy et al., 2015). In line with this, we have found that retigabine-induced vasodilation is increased in PAs from pulmonary hypertensive animals and this is associated with an increased contribution of Kv7 channels to the net K^+^ current present in pulmonary arterial myocytes (unpublished data from the author’s laboratory).

Altogether, these findings suggest that Kv7 channels play a protective role in the pulmonary circulation limiting pulmonary vasoconstriction, and their activation may represent a promising therapeutic strategy in pulmonary hypertension.

## Kv7 Channels: New Pharmacological Targets in Respiratory Diseases?

In addition to their well-established role in neural and cardiac tissue, studies over the last 20 decades have evidenced the expression and the plethora of cellular functions of Kv7 channels in numerous cell types. Thus, Kv7 channels are now recognized as playing relevant physiological roles in many tissues, which has encouraged the search for the therapeutic potential of Kv7 channel modulators in many diseases including those affected the lung. As stated in the previous section activation of Kv7 channels has been proposed to provide beneficial effects in a number of lung conditions. Therefore, Kv7 channel openers/enhancers or drugs acting partly through these channels could be proposed as bronchodilators, expectorants, antitussives, chemotherapeutics, and pulmonary vasodilators, as summarized below.

Flupirtine and retigabine are structurally related Kv7.2–7.5 channel activators. The discovery of Kv7 channels came later than the extensive use of flupirtine and retigabine as an analgesic and anticonvulsant, respectively, and whose mechanism of action was unknown until then (Schenzer et al., 2005). Retigabine has been shown to increase the open probability and cause a hyperpolarizing shift in the voltage dependence of activation of *KCNQ* channels by binding at the intracellular side of the pore-forming S5 helix around a key tryptophan (Trp) Trp236 in Kv7.2 and analogous residues in Kv7.3, 4, and 5 which is absent in Kv7.1 channels (Schenzer et al., 2005; Lange et al., 2009; Kim et al., 2015). Kim et al. have suggested that the retigabine interaction with this Trp residue depends largely on the formation of a hydrogen bond (Kim et al., 2015).

As the relevant role of Kv7 channels was discovered, the idea that these channels could constitute a new therapeutic strategy became more attractive, leading to investment in the development of new activators. The key residues for retigabine-binding have also been recognized to be also essential for the binding of other compounds such as S1 and BMS-204352; though these last drugs have a greater efficacy enhancing Kv7.4 and Kv7.5 isoforms rather than Kv7.2 or/and Kv7.3 channels (Dupuis et al., 2002; Bentzen et al., 2006). Zinc pyrithione activates the Kv7 channel by interacting with a binding site different to that of retigabine (Xiong et al., 2008). Hence, zinc pyrithione activates all Kv7 channels except for Kv7.3 channels, which is the most retigabine-sensitive. It is worth noting that the active compound in the action of zinc pyrithione on Kv7 channels is zinc, as administration of free zinc also activates these channels by reducing their dependence on PIP_2_ (Gao et al., 2017). The activity of ICA-27243, a drug more potent at activating Kv7.2/7.3 heteromeric channels than Kv7.4 homomeric channels or Kv7.3/7.5 heteromeric channels, is determined by a novel site inside the Kv7 channel voltage-detection domain (Wickenden et al., 2008; Blom et al., 2010). R-L3 is a benzodiazepine that activates Kv7.1 homomeric channels, but is considerably weaker when Kv7.1 is coexpressed with KCNE1 subunit, suggesting that R-L3 and KCNE1 subunits compete for the same interaction site on *KCNQ1* subunits (Salata et al., 1998).

Kv7 channels are currently new therapeutic targets in the field of lung diseases; consequently, there is still much to be learned about their physiological role, regulation, and possible impairment by pathological processes. The specific expression of certain Kv7 channel in different types of cells is of great pharmacological interest, since their selective modulation could provide beneficial therapeutic effects while avoiding the adverse effects associated with other isoforms (Stott et al., 2014). A clear example comes from the previous experience with retigabine and flupirtine, which have been used as analgesic and anticonvulsant, respectively, for many years but were withdrawn from the marked due to their adverse effects including the risk of serious liver injury, pigment changes in the retina and urinary retention (Herrmann et al., 1987; Brickel et al., 2012). While, as mentioned, both drugs activate Kv7.2–Kv7.5, they appear to display greater efficacy for Kv7.2 and Kv7.3 channels over Kv7.4 and Kv7.5 channels, which predominate in smooth muscle. Thus, it is expected that compounds selectively activate the Kv7.4 and/or the Kv7.5 subunit may be used in the treatment of vascular smooth muscle disorders with few neurological side effects. For instance, selective activation of Kv7.4 and Kv7.4/Kv7.5 channels (but not Kv7.1, Kv7.2, and Kv7.2/Kv7.3) has been implicated in the vasorelaxant effects of the Rho kinase inhibitor fasudil (Zhang et al., 2016). Thus, it is likely that, in addition to Rho kinase inhibition, activation of these channels may contribute to the beneficial effects of fasudil in cardiovascular conditions such as hypertension, vasospasm, stroke arteriosclerosis, pulmonary hypertension, and heart failure (Shi and Wei, 2013; Shimokawa and Satoh, 2015). Likewise, it is expected that selective Kv7.2 and Kv7.3 channel enhancers may be used to the treatment of neurological disorders with few adverse effects in smooth muscle cells (Stott et al., 2014; Barrese et al., 2018). Remarkably, Manville et al. have recently reported that the neurotransmitter γ-aminobutyric acid (GABA) and various metabolites (β-hydroxybutyric acid and γ-amino-β-hydroxybutyric acid) directly activate Kv7.3 and Kv7.5 but not Kv7.1, 2, and 4 (Manville et al., 2018). Manville and colleagues found that Trp residues in Kv7.3 (Trp265) and Kv7.5 (Trp270) were required for binding but not sufficient for GABA-induced activation. Thus, while Kv7.2 and Kv7.4 share analogous Trp residues and bind GABA, they are not activated by GABA (Manville and Abbott, 2018). Likewise, the synthetic GABA analog gabapentin, but not pregabalin, was also found to activate Kv7.3 and Kv7.5 (Manville and Abbott, 2018). Very recently, these authors have proposed a model in which the sodium-coupled myo-inositol transporter 1 (SMIT1) interacts with KCNQ2/3 and tunes the channel response to GABA and related metabolites (Manville and Abbott, 2020).

The search for Kv7 activators with a better safety profile than retigabine and flupirtine as well as the identification of novel potential therapeutic applications, has driven the development of a large number of compounds with Kv7 enhancing properties in the last decade (Jepps et al., 2013; Stott et al., 2014; Haick and Byron, 2016; Barrese et al., 2018).

Given the functional roles and ubiquitous expression of Kv7 channels in many tissues, drugs modulating Kv7 channels are presumed to cause off-target effects including alterations of the QT interval at the heart level, systemic hypotension, urinary retention or neuronal effects. This could be largely avoided by using localized drug delivery to the lungs by oral inhalation. Inhaled administration is the route of choice for many drugs (i.e., corticosteroids; anticholinegics, or β2-adrenergic receptor agonists) to treat respiratory diseases. Similarly, targeting lung Kv7 channels specifically using inhaled Kv7 activators may be an innovative approach for the treatment of COPD, cystic fibrosis, pulmonary hypertension, asthmatic events, lung cancer, bronchitis, and pulmonary edema.

Progress in the treatment of respiratory diseases is a global challenge because of its health and socio-economic implications. The optimal management and treatment of these diseases should be aimed at achieving adequate control of the disease, improving the quality of life of patients, and reducing hospitalizations and the enormous financial burden on healthcare systems. Targeting Kv7 channels in the treatment of lung disorders could be a promising therapeutic strategy. Here, we summarized the potential usefulness of Kv channel modulators in treating respiratory diseases.

### Bronchodilators

Asthma and COPD are characterized by hyper-contraction of ASMC attributed to abnormally high concentrations of different bronchoconstrictor agonists and/or increased sensitivity to them, leading to pathological narrowing of the airways (Penn, 2008). K^+^ channels are key regulators of Em in ASMC and their impairment causes membrane depolarization and bronchoconstriction (Hall, 2000). Among them, Kv7 channels contribute significantly to the negative resting Em in ASMC (Brueggemann et al., 2011). By acting on a different target, Kv7 channel openers offer the potential of being combined with standard bronchodilators such as β2-adrenergic receptor agonists and antimuscarinics as an adjuvant therapy to increase the efficacy or overcome desensitization (Brueggemann et al., 2014b).

### Expectorants

The function of Kv7.1 together with KCNE1/E2/E3 subunits seems to play a key role in secretions in airway epithelial cells. The co-activation of Cl^−^ channels in the luminal surface and K^+^ channels in the basolateral membrane in AEC is an essential requirement for Cl^−^ secretion (Frizzell and Hanrahan, 2012). The activation of Kv7.1 channels has an expectorant effect provoking or promoting the expulsion of accumulated bronchial secretions and the productive cough helping to airway clearance (Mall et al., 2000; Al-Hazza et al., 2016; Kroncke et al., 2016; Hynes and Harvey, 2019). Kv7.1 channel openers or drugs that indirectly activate Kv7.1 channels might be beneficial in the treatment of bronchitis, COPD, cystic fibrosis and asthma (Boucherot et al., 2001; Athanazio, 2012; Hynes and Harvey, 2019).

### Antitussives

Stimulation of airway C-fibers can be activated by inflammatory factors leading to the impulse to unproductive cough, dyspnea, mucus secretion and bronchoconstriction (Mazzone and Undem, 2016). Activation of Kv7.3 channels in VACF by retigabine has been shown to increase the M-current amplitude and to inhibit unproductive coughing (Sun et al., 2019). Thus, Kv7 activators could be useful as chronic coughing relievers in pulmonary inflammatory diseases.

### Chemotherapy

Although, the role of Kv7 channel modulators as chemotherapy agents has been only barely studied, flupirtine has been shown to attenuate the resistance to gefitinib in a human lung cancer cell line (Choi et al., 2017). The anti-proliferative effect of flupirtine has also been proposed as potentially beneficial in other types of cancer (Lee et al., 2014). Additional studies are required to evaluate the potential of Kv7 channel activators with selectivity for particular isoforms as chemotherapy agents.

### Pulmonary Vasodilators

K^+^ channels are also key regulators of cellular Em in PASMCs, controlling the activity of voltage-dependent Ca^2+^ channels and, subsequently, pulmonary artery smooth muscle contraction. Pulmonary vascular tone is controlled by the interaction of different circulating or locally released vasoactive factors with vasoconstrictor or vasodilating properties. Acute modulation of the activity of K^+^ channels has been extensively shown to contribute to the vascular effects of these vasoactive factors. Thus, different vasodilators such as nitric oxide, prostacyclin and calcitonin gene-related activate several K^+^ channels and, conversely, vasoconstrictors as serotonin, angiotensin II, thromboxane A_2_, noradrenaline and endothelin-1 have been shown to inhibit K^+^ channels (Cogolludo et al., 2007; Stott et al., 2014). With such a crucial role in modulating and maintaining pulmonary arterial tone, changes in function and expression of K^+^ channels during the development and pathogenesis of pulmonary hypertension results in aberrations of normal physiological functions. Consequently, K^+^ channels openers are presumed to have a beneficial therapeutic effect, especially if their action can be directed to the pulmonary circulation (Gurney et al., 2010). Up to the present time, the availability of drugs modulating Kv7 channels has been relatively scarce. Advances in the understanding of the role of Kv7 channels in the pulmonary vasculature and the development of new selective drugs for these channels indicate that they may represent a useful therapeutic target for the treatment of pulmonary arterial hypertension. As detailed above, different studies have provided evidence that treatment with Kv7 channel activators such as flupirtine, retigabine, or fasudil is beneficial in different pulmonary hypertension models (Morecroft et al., 2009; Duong-Quy et al., 2013; Sedivy et al., 2015; Zhang and Wu, 2017). In addition, Kv7 channel activation has been shown to contribute to the pulmonary vasodilating effect induced by drugs approved for the pulmonary hypertension treatment such as nitric oxide or riociguat (Mondejar-Parreño et al., 2019).

## Conclusions

In the last decade, a significant progress in the understanding of the role of Kv7 channels in lung physiology has been made. The recognition of the involvement of these channels in cellular functions of crucial relevance in the normal physiology of the lung, such as the control of water and salt transport across the epithelial cell membrane, the excitability of airway afferent nerves, and the regulation of airway and lung vascular smooth muscle tone, makes them attractive therapeutic targets in lung diseases. In this regard, deciphering the involvement and importance of specific Kv7 subunits in these cellular functions will lead to a significant advance that may constitute the basis for the therapeutic potential of selective Kv7 channel modulators in lung diseases.

## Author Contributions

GM-P, FP-V, and AC wrote the manuscript. All authors contributed to the article and approved the submitted version.

## Conflict of Interest

The authors declare that the research was conducted in the absence of any commercial or financial relationships that could be construed as a potential conflict of interest.
